# Iron biofortification as a promising strategy to improve productivity and nutritional value of *Arthrospira platensis* (spirulina)

**DOI:** 10.1038/s41598-026-40520-8

**Published:** 2026-02-21

**Authors:** Fatemeh Gholizadeh, Fatemeh Zarinkamar

**Affiliations:** Department of Plant Biology, Faculty of Biological Sciences, Tarbiat Modares University, Tehran, Iran

**Keywords:** Artrospira platensis, growth performance, iron biofortification, biochemical composition, nutritional value, phycobiliproteins, polyunsaturated fatty acids, Biochemistry, Biotechnology, Environmental sciences, Microbiology, Plant sciences

## Abstract

*Arthrospira platensis* (spirulina) is widely recognized as a nutrient-dense cyanobacterium. The present study is aimed at investigating the role of iron biofortification on the productivity and nutritional value of the biomass. The cyanobacterium was grown in the Zarrouk’s medium supplemented with certain iron concentrations (2, 8, 16, 32, and 64 mg L^− 1^). The biomass growth was assessed by measuring specific growth rate and yield and then analyzed for iron uptake, protein, carbohydrates, fatty acids, phycobiliproteins, and phenolics by different assays using UV/Vis spectroscopy and inductively coupled plasma optical emission spectrometry. Increasing iron levels in the media resulted in higher growth rate, biomass density, protein, carbohydrates, and reactive oxygen species. We only observed significant changes in fatty acids, phycobiliproteins, and phenolics at iron levels > 16 mg L^− 1^. The highest unsaturated fatty acids and phycocyanin were observed at the moderate iron levels (~ 32 mg L^− 1^); while higher values increased phenolics, particularly benzoic and gallic acids (~ 2.5- to 7-fold). The moderate iron level is critical to increase biomass productivity, unsaturated fatty acids, and phycobiliproteins, to control anti-nutritional phenolics, and to minimize iron-induced oxidative stress.

**Supplementary Information:**

The online version contains supplementary material available at 10.1038/s41598-026-40520-8.

## Introduction

Cyanobacteria are known as a promising source of functional foods. Among these microorganisms, *Arthrospira platensis*, a filamentous gram-negative cyanobacterium, first isolated by Turpin in 1827^1,2^, was recognized by the World Health Organization as a “superfood”^3^. *A. platensis* is consumed worldwide as a dietary supplement due to its exceptional nutritional profile with the commercial name of spirulina for the dried biomass. *A. platensis* comprises a high protein content (up to 70% of its dry weight) including all essential amino acids. It has a rich array of polyunsaturated fatty acids (PUFAs), notably gamma-linolenic acid (36% of PUFAs)^4^. Spirulina biomass is a valuable source of micronutrients including essential vitamins and minerals. The presence of bioactive compounds such as phycocyanin, chlorophyll, and carotenoids in *A. platensis* contributes to its potent antioxidant and anti-inflammatory properties^5,6^. *A. platensis* is an iron hyperaccumulator. The iron bioavailability of this biomass is almost 60% higher than that of conventional ferrous sulfate supplements^3,7,8^.

Iron deficiency is a significant risk factor for anemia, cardiovascular disease, cancer, and impaired neurodevelopment^9^. Iron deficiency anemia (IDA) remains one of the most widespread and common nutritional disorders globally. This high prevalence of IDA is often linked to unbalanced dietary iron intake, especially in developing countries. If left unaddressed, IDA can lead to serious negative health outcomes such as low productivity, high morbidity and mortality, developmental delays, and stunted growth^2^. The most common interventions for addressing IDA include direct iron supplementation and food fortification. For example, daily oral supplementation of ≥ 30 and 100 mg ferrous iron was recommended between meals for healthy women and those with IDA, respectively^10^. The iron fortification of foods is considered the most practical and cost-effective long-term solution. However, this approach faces several challenges such as undesirable taste and color and more importantly degradation of valuable nutrients. On the other hand, the effectiveness of iron fortification can significantly be compromised by anti-nutritional factors such as polyphenols which might reduce iron bioavailability^2,11^. *A. platensis* is a promising candidate for iron biofortification considering its exceptional natural iron content. Research indicates that daily supplementation with 1 g spirulina over eight weeks can significantly improve anemia-related symptoms in adults. Spirulina demonstrates high digestibility and lacks significant anti-nutritional factors, while exhibiting superior iron bioavailability compared to beef meat^1,12^. Thus, enriching and biofortifying *A. platensis* with iron sounds a promising solution to address iron deficiency^13^.

Generally, iron exists in two chemical forms including ferric ion and ferrous ion. In a neutral aqueous environment, the ferric ion form is thermodynamically more stable and has very low solubility. The ferric ions precipitate as insoluble oxides or hydroxides in the culture media, which is largely unavailable to cyanobacteria. Besides, the ferric ion may absorb other essential elements and lower their availability too. In contrast, the ferrous ions have higher solubility, but they are thermodynamically unstable and easily oxidized to ferric ions, while the water-soluble form (or ferrous ion) is required for cellular uptake and consequent metabolic activities^2^.

Iron plays a pivotal role in the growth and development of cyanobacteria. Iron is critical to metabolic processes such as photosynthetic electron transfer, nitrogen fixation, and cellular respiration^14,15^. *A. platensis* possesses a remarkable ability to take up iron metals from the culture media through both biosorption and bioaccumulation mechanisms. Recent studies have demonstrated that increasing iron concentration in the culture medium can significantly boost the iron uptake of the biomass without any negative effect on cyanobacterial growth and productivity^2^. The proton-active functional groups in the cell wall, together with the polymeric substances in the exopolysaccharide layer, significantly enhance the adsorption of the positively charged iron ions^16^. Iron’s cross-talk with other macro-and micro-nutrients in the plant cells can also significantly affect their concentration and consequently the governing metabolic processes in the cells^17^. For example, Fe interacts antagonistically with other nutrients such as phosphate, Zn and Mn to keep nutrient homeostasis in a cell^17^. Iron deficiency promotes higher cellular uptake and accumulation of these nutrients.

Therefore, the present study aimed at exploring the impact of varying iron concentrations on the biomass productivity of *A. platensis*, iron accumulation, the contents of carbohydrates and proteins, and the profile of essential fatty acids and phycobiliproteins. In addition, we assessed the oxidative status and phenolics profile. Specifically, we examined the effect of excess iron on the phenolic acid profiles, hydrogen peroxide, and antioxidant biomarker malondialdehyde (MDA). This investigation is crucial to optimize nutritional value by iron biofortification while ensuring iron bioavailability and preventing oxidative stress. To the best of our knowledge, this is the first report regarding the impact of iron biofortification of *A. platensis* on the nutritional value of spirulina.

## Materials and methods

### Cyanobacterium and culture materials

*Arthrospira platensis* AFC 006 was obtained from the cell bank of Afagh Zist Collection (Tehran, Iran). The culture materials in Zarrouk’s medium were bought from Merck (Darmstadt, Germany) in reagent grade.

### Analytical standards and reagents

For chemical assays, the standards and reagents including Folin-Ciocalteu reagent, nitric acid, calcium chloride, sulfuric acid, ethanol, methanol, acetone, acetonitrile, acetic acid, hydrogen chloride, n-hexane, sodium carbonate, cinnamic acid, para-coumaric acid, gallic acid, hydroxybenzoic acid, ferulic acid, salicylic acid, benzoic acid, potassium phosphate, potassium iodide, L-methionine, sulfosalicylic acid, and L-proline were procured from Merck (Darmstadt, Germany). The other ones including thiobarbituric acid, glacial acetic acid, trichloroacetic acid, riboflavin, ninhydrin, nitroblue tetrazolium, and toluene were purchased from Sigma-Aldrich (St. Louis, MO, USA). All these chemicals were of analytical grade.

### *A. platensis* cultivation and iron biofortification

*A. platensis* was cultivated in the modified Zarrouk’s medium with initial pH 9.5^18^. The medium composed of 16.8 g NaHCO₃, 0.5 g K₂HPO₄, 2.5 g NaNO₃, 1.0 g K₂SO₄, 1.0 g NaCl, 0.2 g MgSO₄0.7 H₂O, 0.04 g CaCl₂, 0.01 g FeSO₄0.7 H₂O, and 0.08 g Na-EDTA in one liter solution, plus 1 mL micronutrient solution which includes 2.86 g H₃BO₃, 1.81 g MnCl₂0.4 H₂O, 0.222 g ZnSO₄0.7 H₂O, 0.079 g CuSO₄0.5 H₂O, and 0.007 g Na₂MoO₄.H₂O per liter solution. The cyanobacterium was grown in 1 L glass bottle under continuous aeration with compressed air at 0.7 L min^− 1^. The culture temperature was set at 30 °C and the light was provided by cool natural white fluorescent lamps at an intensity of 150 µmol photons m^− 2^ s^− 1^ and a light/dark photoperiod of 16/8. For iron biofortification, the culture medium was supplemented with excess FeSO₄0.7 H₂O, which is the recommended iron salt in the ferrous ion form for biofortification purpose^2^. In this work, we studied the effect of iron at varying concentrations of 2 (control), 8, 16, 32, and 64 mg Fe L^− 1^ in the first day of cultivation to ensure immediate access to cyanobacterium. This range of iron concentration was chosen based on preliminary tests and literature review^1,2^. Indeed, iron levels higher than the upper range tend to conglomerate and cannot pass through filters. The culture period lasted 14 days, then the culture was halted and the cells were harvested for further assays.

### Assessment of cyanobacterial growth

We measured cell density, growth rate, doubling time, and biomass productivity. These measurements provide a quantifiable metric of the growth performance. The cell density of *A. platensis* was determined every 48 h by measuring optical density (OD) at 560 nm using a UV/vis spectrophotometer (MAPADA, China). The maximum specific growth rate (µ_max_) was calculated using OD measurements at the beginning (t_1_) and end (t_2_) of the exponential growth phase in day as follows^19^:

1 $$\:{\mu\:}_{max}\left({d}^{-1}\right)=\left(ln{OD}_{2}-ln{OD}_{1}\right){\left({t}_{2}-{t}_{1}\right)}^{-1}$$

The doubling time (D_t_) was calculated by the specific growth rate (µ _max_) using the following Equation 2^20^:

2 $$\:{D}_{t}\left({d}^{-1}\right)=Ln\left(2/{\mu\:}_{max}\right)$$

The OD was correlated with dry weight (DW) biomass concentration. The maximum dry biomass (*X*_*max*_) was determined at the end of the cultivation period. A 20 mL sample of the culture was harvested by filtration through a 30-micron nylon mesh and then the biomass was washed thoroughly with double distilled water to remove residual medium. The collected biomass was then dried in an air-circulated oven at 65 °C for 24 h to ensure complete moisture removal^21^. The DW-to-FW conversion factor is 0.357.

The maximum biomass productivity (*P*_*max*_) was estimated through Eq. 3 to assess the cyanobacteria’s ability in converting nutrients and light into biomass over time.

3 $$\:{P}_{max}\left(g{l}^{-1}{d}^{-1}\right)=({X}_{t}-{X}_{0})/(t-{t}_{0})\:$$

Where *X*_*t*_ ​ and *X*_*0*_ ​represent the biomass concentrations (g L^-1^ at the end (*t*) and beginning (t_0_) of the cultivation period, respectively.

### Tracing total iron concentration in the *A. platensis* biomass

The dried biomass (0.1 g) was mixed with 10 mL concentrated nitric acid (HNO₃) in a microwave digestion vessel at 180 °C for 15 min. We used a microwave-assisted acid digestion protocol to ensure thorough break down of the organic matrix and accurate mineral quantification. After digestion, the solution was cooled to room temperature to prevent pressure build-up during vessel opening. The digested sample was diluted to a final volume of 50 mL using sterile double distilled water and filtered through a 0.45 μm membrane filter to remove any particulate matter, ensuring clarity for analysis. The prepared solution was analyzed using inductively coupled plasma optical emission spectrometry (ICP-OES) with a Thermo Scientific iCAP 6000 series model (Thermo Fisher Scientific, Waltham, MA, USA) under optimized operating conditions at 1.2 kW radiofrequency power, 0.5 L min^-1^ nebulizer gas flow, 50 rpm pumping speed, and no stabilization time. Iron concentration was read at 259.94 nm, associated with iron’s emission wavelength spectra.

### Phycobiliprotein extraction and assessment

The phycobiliproteins (PBPs) were extracted from *A. platensis* by the modified method^22^. *A. platensis* culture (1.8 mL) was centrifuged at 10,000 rpm for 5 min. The biomass was then resuspended in a 1.5% (W/V) CaCl_2_ solution to enhance cell membrane permeability and the mixture underwent four freeze/thaw cycles to ensure the release of PBPs into the solution. Thereafter, the mixture was homogenized for 2 min to further break down any remaining cell structures and ensure a uniform extract. The homogenate was centrifuged at 4000 rpm and 20 °C for 10 min. The absorbance of the supernatant was measured at 562, 615 and 652 nm using the spectrophotometer to quantify three PBPs, namely phycocyanin (PC), allophycocyanin (APC), and phycoerythrin (PE), respectively. The concentrations were determined using the following correlations^23^:

4 $$\:\left[PC\right]=({OD}_{615}-0.474{OD}_{652})/5.34$$

5 $$\:\left[APC\right]=({OD}_{652}-0.208{OD}_{615})/5.09$$

6 $$\:\left[PE\right]=({OD}_{562}-2.41PC-0.849APC)/9.62$$

The purity and yield of the extracted PBPs were evaluated through spectrophotometric analysis. The specific absorbance of PBPs at 620 nm relative to the absorbance of total protein at 280 nm in the sample, offers a measure of purity of the extract:

7 $$\:purity={OD}_{620}/{OD}_{280}$$

The yields of individual PC, APC, and PE were calculated using their respective concentrations (C) and extract volume (V) relative to biomass dry weight (DW) as follows:

8 $$\:Yield\:\left(mg\:{g}^{-1}\right)=C.V/DW$$

### Total protein quantification

The protein content in the biomass was determined by the Bradford assay^24^. The fresh biomass (0.2 g) was homogenized in Tris-HCl buffer (pH 6.8). The homogenate was then centrifuged at 13,000 rpm and 4 °C for 20 min to separate the soluble proteins from the cellular debris. A 100 µL aliquot of the extract was gently vortexed with 1 mL Bradford reagent and then incubated at room temperature for 20 min to allow optimal color development. The absorbance of the solution was read at 595 nm using the spectrophotometer and the results were expressed in mg protein per g biomass DW.

### Total carbohydrate quantification

The total carbohydrates of *A. platensis* was determined by the phenol-sulfuric acid method^25^. The fresh biomass (0.1 g) was homogenized in 3 mL distilled water. The mixture was then centrifuged at 5,000 rpm for 20 min. A 500 µL supernatant was mixed with 500 µL phenol solution (5%), followed by rapid addition of 1.5 mL H_2_SO_4_ (72%). The mixture was vortexed and incubated at room temperature for 10 min before being heated in a boiling water bath at 90 °C for 5 min. After cooling to room temperature, the absorbance was measured at 490 nm using the spectrophotometer. The total carbohydrates were quantified by a calibration curve method using glucose as standard and the results were expressed in mg total carbohydrate per g biomass DW.

### Assessment of fatty acids

Fatty acid analysis was conducted using gas chromatography (GC). The dried biomass (0.1 g) was dissolved in 2 mL sulfuric acid-methanol solution (2.5% v/v) and then incubated in a water bath at 80 °C for 1 h. After cooling, the solution was mixed with 1 mL n-hexane and 1.5 mL sodium chloride solution (1% w/v). The mixture was centrifuged at 4000 rpm for 5 min. The supernatant was collected and transferred to a 2 mL microtube, after which an additional 1 mL n-hexane was added and the mixture was centrifuged for phase separation. The final supernatant was then separated and the solvent was evaporated in air. Finally, 0.5 mL isooctane was added to the methyl ester residues, and the solution (1 µL) was injected into the GC-2014 (Shimadzu, Japan) equipped with a flame ionization detector (FID) and a SP-2330 fused silica capillary column RTX-wax (30 m, 0.25 mm, 0.25 μm). The system temperature was programmed from initial temperature of 70 °C, ramping up to 250 °C/300°C, holding for 30 min. Nitrogen was used as the carrier gas at a flow rate of 1.26 mL min^-1^, with a split ratio of 100:1. Fatty acids were identified by comparing the retention times with authentic standards in the GC-MS database. The fatty acid concentration was determined based on the peak areas using a linear calibration curve (R² = 0.99) in the HP 3365 ChemStation program. The results were confirmed within a 95% confidence interval and expressed as the percentage of total fatty acids. Every sample was analyzed in triplicate (*n* = 3) to ensure data accuracy and reproducibility.

### Assessment of total phenolics

The phenolic compounds of the biomass were analyzed by an optimized Folin-Ciocalteu method^26^. Briefly, 0.1 g fresh biomass was suspended in 5 mL methanol (80%). The mixture was then centrifuged at 12,000 rpm for 15 min. A 100 µL aliquot of the supernatant was combined with 500 µL double distilled water and 100 µL Folin-Ciocalteu reagent, followed by vortex mixing and incubation at room temperature for 6 min. Then, 1 mL Na_2_CO_3_ solution (7%) was added, and the mixture was incubated in darkness for 90 min. The absorbance was measured at 760 nm using the spectrophotometer. Total phenolic content (TPC) was quantified using a calibration curve with gallic acid as standard and expressed as mg gallic acid equivalent per gram dry biomass (mg GAE g^-1^ DW).

### Phenolic acid analysis

The fresh biomass (0.5 g) was homogenized in 4 mL methanol and then centrifuged at 12,000 rpm for 15 min. The supernatant was collected and evaporated to dryness. The dried extract was resuspended in 4 mL acetonitrile, and mixed with 3 mL n-hexane for phase separation. The lower phase was carefully collected and dried under nitrogen gas to prevent oxidation. The final residue was dissolved in 500 µL methanol and filtered through a 0.22 μm syringe filter before analysis. The HPLC analysis was conducted on a C18-ODS3 column (Perfectsil Target ODS-3 (5 μm), 250 mm × 4.6 mm; MZ Analysentechnik, Germany) using a mobile phase composed of a gradient mixture of methanol and acidified water (2% acetic acid in deionized water) at a flow rate of 1 mL min^-1^. The phenolic acids were detected by a UV detector set at 278 nm and 300 nm, with the column temperature maintained at 25 °C. The phenolic acids were identified by comparing the retention times with those of the standards and quantified by the peak areas using the available correlations^27^.

### Assessment of the oxidative status

The oxidative status was determined by monitoring hydrogen peroxide (H_2_O_2_) content as well as malondialdehyde (MDA) in the biomass. In brief, fresh biomass (0.1 g) was homogenized in 2 mL trichloroacetic acid solution (0.1%) while remaining on ice, followed by centrifugation at 12,000 rpm and at 4 °C for 15 min.

The H_2_O_2_ content was measured using the colorimetric method^28^. A 0.5 mL supernatant was combined with 0.5 mL solution of potassium iodide solution (1 M) in potassium phosphate buffer (0.1 M, pH 7.0). The absorbance was then read at 390 nm using the spectrophotometer. The H_2_O_2_ concentration was quantified against a standard curve.

The MDA, a marker of lipid peroxidation, was measured by mixing a 500 µL supernatant with 1 mL TCA/TBA reagent. This mixture was boiled for 30 min to promote the reaction and then cooled on ice to halt the reaction. The mixture was centrifuged at 13,000 rpm for 10 min. The supernatant’s absorbance was read at 532 nm and 600 nm (non-specific absorption) using the spectrophotometer^29^. The MDA concentration was estimated using an extinction coefficient of 155 mM^-1^ cm^-1^ and expressed as mg per g dry weight.

### Statistical analysis

All the experiments were conducted in triplicates and the data was reported as means ± standard deviation (*n* = 3). To compare the set of the means, we used one-way analysis of variance (ANOVA) at least significant difference of 5% using Duncan’s multiple range test in the SPSS version 26.0 environment.

## Results

### Analysis of cyanobacterial growth performance

The cyanobacteria showed a normal growth in all culture media without any lag phase (see supplementary material). To understand growth performance across different iron treatments, the specific growth rate (µ_max_) was measured. The continuous increase in iron from 2 (control) to 64 mg Fe L^− 1^ caused a significant increase in the maximum specific growth rate (µ_max_) and dry biomass (X _max_), and continuous decrease in doubling time (Dt), Table 1. The µ_max_ progressively increased with higher iron levels, reaching a maximum 0.213 d^− 1^ at 64 mg L^− 1^. Likewise, the shortest Dt (3.20 d) was observed at 64 mg L^− 1^, whereas the control condition (2 mg L^− 1^) exhibited the longest Dt (3.85 d). A shorter doubling time indicates faster growth, which aligns with the observed increase in growth rate at higher iron levels. The maximum biomass was peaked at 1.511 g L^− 1^ with 32 mg Fe L^− 1^ so that no significant change was observed at higher iron levels 64 mg L^− 1^. There were no significant changes in the biomass productivity (P _max_) across all treatments.

**Table 1:** Effect of varying levels of iron supplementation on the specific growth rate, doubling time, maximum biomass and biomass productivity of *A. platensis*.

Iron concentration (mg L^-1^)	µ_max_ (d^-1^)	D_t_ (d)	X _max_ (g L^-1^)	*P*_max_ (g L^-1^ d^-1^)
control^*^	0.180^c^ ± 0.004	3.85^d^ ± 0.10	1.096 ^b^ ± 0.10	0.13 ^ns^ ± 0.008
8	0.192^bc^ ± 0.006	3.61^c^ ± 0.13	1.106 ^b^ ± 0.04	0.13 ^ns^ ± 0.014
16	0.195^bc^ ± 0.001	3.55^bc^ ± 0.02	1.165 ^b^ ± 0.15	0.14 ^ns^ ± 0.001
32	0.205^ab^ ± 0.008	3.39^ab^ ± 0.14	1.511 ^a^ ± 0.14	0.16 ^ns^ ± 0.015
64	0.213^a^ ± 0.009	3.20^a^ ± 0.11	1.450 ^a^ ± 0.13	0.15 ^ns^ ± 0.02

*The control concentration is equal to 2 mg Fe L-1 in the Zarrouk’s medium. Values are means ± standard deviation (*n* = 3). Different superscript letters show significant differences at *p* < 0.05.

### Biochemical composition under iron supplementation

**Fig. 1:**
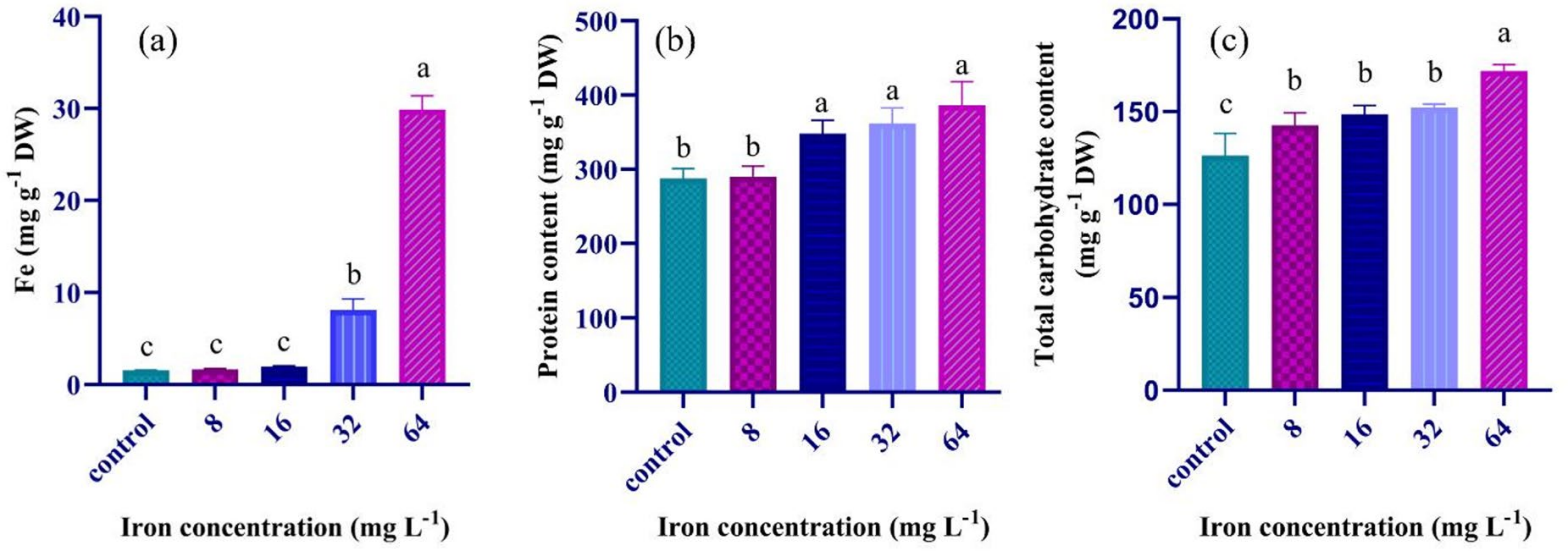
Effects of varying levels of iron supplementation to the culture medium on the total adsorbed iron by the cyanobacterium (**a**), total protein (**b**) and total carbohydrate (**c**) contents. The superscript letters show statistically significant differences at *p* < 0.05. The control is equal to 2 mg Fe L^− 1^ in the Zarrouk’s medium.

Analysis of the iron content in *A. platensis* biomass revealed a dose dependent relationship with iron level in the culture medium (Fig. 1a). At lower iron levels (2, 8, and 16 mg L^-1^, the total iron content remained relatively stable, ranging from 1.56 to 1.94 mg g^-1^ DW; while there was a significant surge in the iron content at higher concentrations: 8.10 mg g^-1^ DW at 32 mg Fe L^-1^ and 29.87 mg g^-1^ DW at 64 mg Fe L^-1^.

The protein content of *A. platensis* showed a clear upward trend in response to increasing iron concentration (Fig. 1b). At the control iron concentration (2 mg L^− 1^), total protein content measured 287.76 mg g^− 1^ DW, while at the highest concentration (64 mg L^− 1^), it significantly increased (34%) to 386.41 mg g^− 1^ DW.

The soluble carbohydrate content also showed a positive correlation with iron concentration, as evidenced by a significant increase in the soluble carbohydrates with iron supplementation from 8 to 64 mg L^− 1^ (Fig. 1c). The highest content (171.90 mg g^− 1^ DW) was observed at 64 mg L^− 1^.

### Effect of iron biofortification on the oxidative status

**Fig. 2:**
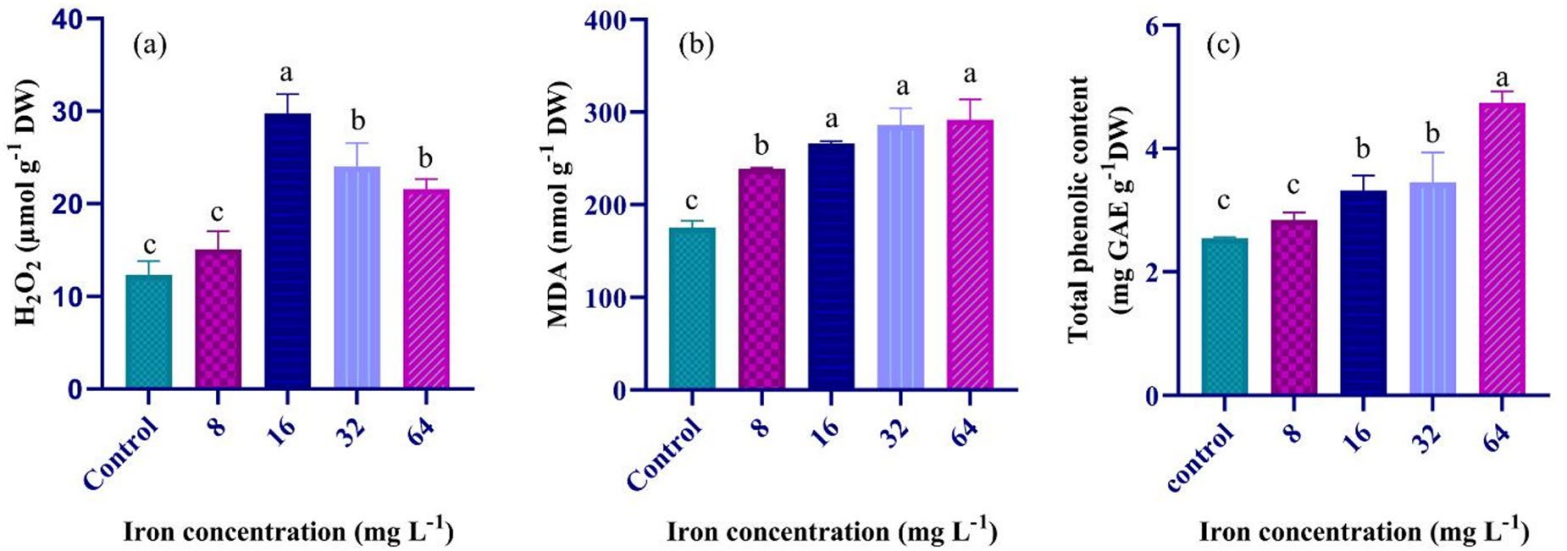
Effects of iron fortification on the H₂O₂ content (**a**), MDA (**b**), and total phenolics (**c**) of *A. platensis* upon cultivation in the medium supplemented with varying iron concentrations (control, 8, 16, 32, and 64 mg Fe L^-1^. The bars represent mean ± standard deviation (*n* = 3). Different letters above the bars show statistically significant differences at *p* < 0.05. The control is equal to 2 mg Fe L^-1^ in the Zarrouk’s medium.

The H₂O₂ and MDA in *A. platensis* was assessed across certain iron levels to evaluate oxidative status. The H₂O₂ level increased significantly to 29.76 µmol g^-1^ DW at an iron concentration of 16 mg L^-1^(Fig. 2a), indicating enhanced oxidative stress due to increased accumulation of reactive oxygen species (ROS). However, at higher iron concentrations (32 mg L^-1^ and 64 mg L^-1^, H₂O₂ levels decreased slightly (almost 23%). Consistently, the MDA level increased significantly with iron concentration, indicating enhanced oxidative status (Fig. 2b). Specifically, MDA levels rose almost 67%, from 175.13 nmol g^-1^ DW at 2 mg L^-1^(control) to 291.88 nmol g^-1^ DW at 64 mg L^-1^.

### Fatty acid profile under iron biofortification

Both saturated and unsaturated fatty acids of *A. platensis* were significantly changed under varying iron concentrations in the culture medium (Table 2). Among the saturated fatty acids, the palmitic acid (C16:0) showed higher levels at low iron concentrations, with the highest level (51.45%) at 8 mg L^− 1^ iron treatment; while the palmitoleic acid (C16:1) showed a marked increase at higher iron concentrations of 32 and 64 mg L^− 1^. Similarly, the stearic acid (C18:0) decreased with an increase in iron concentration whereas the highest level (1.43%) was observed at moderate concentration of 16 mg L^− 1^. Notably, the oleic acid (C18:1) also decreased upon medium enrichment with higher iron concentration. The oleic acid levels were significantly lower at iron concentrations above 64 mg L^− 1^, suggesting that excess iron may suppress its biosynthesis. The linoleic acid (C18:2) and linolenic acid (C18:3) also showed notable variations, both peaked at 32 mg L^− 1^ and then reduced at 64 mg L^− 1^.

**Table 2:** Fatty acid profile of *A. platensis* biomass cultured under varying iron concentrations.

Fatty acid (% of total fatty acids)	Iron concentration (mg L^-1^)				
	Control	8	16	32	64
Undecanoic (C11:0)	5.52 ^ns^ ± 0.68	6.67 ^ns^ ± 0.18	5.91 ^ns^ ± 0.65	5.23 ^ns^ ± 1.84	6.36 ^ns^ ± 1.34
Palmitic acid (C16:0)	49.59 ^ns^ ± 1.39	**51.45** ^**ns**^ **± 0.66**	50.27 ^ns^ ± 0.95	45.84 ^ns^ ± 0.51	50.93 ^ns^ ± 2.25
Palmitoleic acid (C16:1)	3.07 ^b^ ± 0.23	3.08 ^b^ ± 0.26	3.63 ^b^ ±0.30	**4.61** ^**a**^ **± 0.33**	4.56 ^a^ ± 0.94
Stearic acid (C18:0)	1.25 ^a^ ± 0.20	1.38 ^a^ ± 0.12	**1.43** ^**a**^ **± 0.53**	1.07 ^ab^ ± 0.26	0.54 ^b^ ± 0.19
Oleic acid (C18:1)	6.54 ^a^ ± 0.64	4.67 ^b^ ± 0.86	4.60 ^b^ ± 0.90	**4.75** ^**b**^ **± 0.41**	4.16 ^b^ ± 0.43
Linoleic acid (C18:2)	19.80 ^a^ ± 0.32	19.27 ^a^ ± 0.30	19.02 ^ab^ ± 0.22	**19.54** ^**a**^ **± 1.02**	18.30 ^b^ ± 0.60
Linolenic acid (C18:3)	14.22 ^b^ ± 0.96	13.46 ^b^ ± 0.87	15.13 ^b^ ± 1.05	**18.95** ^**a**^ **± 1.32**	15.41 ^b^ ± 2.44

Data are presented as mean ± standard deviation (*n* = 3). Different letters within each column indicate statistically significant differences at *p* < 0.05. The control is equal to 2 mg Fe L^-1^ in the Zarrouk’s medium.

### Phycobiliprotein profile under iron biofortification

The purity index and yield of three phycobiliproteins were measured under different iron concentrations (Table 3). The contents of PC and APC were increased up to 59.84 µg mL^-1^ and 34.99 µg mL^-1^, respectively, upon supplementing the culture media with 16 mg Fe L^-1^. At higher levels, we observed a decrease in these two PBPs. The maximum PE content (4.10 µg mL^-1^) was observed at moderate level of 8 mg Fe L^-1^. The contents of all PBPs were significantly declined at higher levels of iron, suggesting that excessive iron negatively affects the PBP synthesis. The yield of the phycobiliproteins were showed a similar trend. Consistently, the purity index increased from 0.65 in the control sample to 0.86 in the biomass exposed to 16 mg Fe L^-1^, but then declined at higher iron levels (Table 3).

**Table 3:** PC, APC and PE contents of *A. platensis* biomass at different iron concentrations in the culture media.

Iron concentration (mg L^− 1^)	PC (µg mL^− 1^)	APC (µg mL^− 1^)	PE (µg mL^− 1^)	Purity index	Yield PC (mg g^− 1^ DW)	Yield APC (mg g^− 1^ DW)	Yield PE (mg g^− 1^ DW)
control	49.72^b^ ± 2.32	27.99^c^ ± 0.49	3.73^ab^ ± 0.24	0.65^b^ ± 0.04	137.28^ab^ ±19.25	77.09^b^ ± 8.07	10.23^a^ ± 0.34
8	51.65^a^ ± 3.95	30.08^b^ ± 1.53	4.10^a^ ± 0.24	0.81^a^ ± 0.01	139.90^ab^ ± 5.65	81.52^ab^ ± 1.51	11.15^a^ ± 1.11
16	59.84^a^ ± 3.36	36.61^a^ ± 0.59	3.71^ab^ ± 0.27	0.86^a^ ± 0.05	156.38^a^ ±25.80	95.51^a^ ± 13.55	9.64^a^ ± 1.15
32	55.59^a^ ± 0.06	34.99^a^ ± 1.07	3.45^b^ ± 0.44	0.81^b^ ± 0.01	118.85^b^ ±10.55	69.84^bc^ ± 6.69	6.89^b^ ± 1.09
64	42.83^c^ ± 0.61	27.48^c^ ± 0.86	2.61^c^ ± 0.20	0.69^b^ ± 0.005	89.23^c^ ± 9.62	57.21^c^ ± 5.75	5.43^b^ ± 0.67

Values are presented as means ± standard deviation (*n* = 3). Different superscript letters within each column indicate statistically significant differences at *p* < 0.05. The control is equal to 2 mg Fe L^-1^ in the Zarrouk’s medium.

**Table 4:** the profile of phenolics in *A. platensis* under different iron concentrations. Values are mean ± standard deviation (*n* = 3).

Phenolic profile (µg g^-1^ DW)	Iron concentration (mg L^-1^)				
	control	8	16	32	64
Gallic acid	30.88^c^ ± 0.94	38.40 ^c^ ± 0.60	43.11 ^c^ ± 8.77	134.28 ^b^ ± 17.26	210.28 ^a^ ± 11.93
Hydroxy benzoic acid	13.62 ^c^ ± 0.01	13.81^c^ ± 0.85	13.67^c^ ± 0.07	15.49^b^ ± 1.09	19.63^a^ ± 0.03
Ferulic acid	22.36 ^b^ ± 1.05	26.30 ^a^ ± 0.44	26.93 ^a^ ± 0.86	22.76 ^b^ ± 1.01	19.72 ^c^ ± 1.49
Salicylic acid	158.00^ns^ ± 1.97	156.56^ns^ ± 4.35	165.16 ^ns^ ± 17.06	185.04^ns^ ± 30.85	188.67^ns^ ± 28.08
Benzoic acid	73.03 ^b^ ± 10.45	84.28 ^b^ ± 17.08	119.62 ^b^ ± 5.75	198.41 ^a^ ±32.65	199.39 ^a^ ± 49.21
Cinnamic acid	13.33 ^ns^ ±0.24	13.13 ^ns^ ± 0.53	13.14 ^ns^ ± 0.64	14.19 ^ns^ ± 0.06	13.37 ^ns^ ± 0.49

Different letters within each row indicate statistically significant differences at *p* < 0.05; ns indicates no significant difference. The control is equal to 2 mg Fe L^-1^ in the Zarrouk’s medium.

### Effects of various iron concentration on the content and profile of phenolics

Total phenolics in *A. platensis* were measured under varying iron concentrations (Fig. 2c). The total phenolics significantly increased (up to 87%) with iron enrichment. The peak value (4.74 mg GAE g^-1^ DW) occurred at the highest iron concentration 64 mg L^-1^ (Fig. 2c). To gain deeper insight into the association between iron concentration and phenolic compounds in *A. platensis*, HPLC was used to characterize the individual phenolic profiles of biomass grown under iron biofortification at different levels. This technique enabled precise detection and quantification of phenolics. The identified compounds with their relative proportions were presented in Table 4. The analysis revealed notable variations in the accumulation of phenolic acids. The gallic acid, hydroxy benzoic acid, and benzoic acid exhibited marked increases at higher iron concentrations, peaking at 64 mg L^-1^. In contrast, ferulic acid reached its maximum at 8 and 16 mg L^-1^ but declined at elevated iron levels. There was 27% reduction in the content of ferulic acid at 64 mg L^-1^ compared to that of 16 mg L^-1^. Salicylic acid remained relatively stable across treatments, averaged 170.68 ± 12.97 µg g^[-1^ DW. Cinnamic acid level also remained unchanged, averaged 13.43 ± 0.30 µg g^-1^ DW, indicating minimal impact of iron concentration on its production.

## Discussion

*A. platensis*, a cyanobacterium renowned for its high nutritional value, has garnered significant attention as a food supplement due to its exceptional protein content, rich supply of micronutrients, bioactive compounds such as phycocyanin, and essential fatty acids particularly omega-3 and omega-6.

Notably, *A. platensis* is known as an iron hyperaccumulator which makes it particularly promising for addressing iron deficiency and anemia ^2^. Similarly, this study confirmed hyperaccumulation of iron (almost 15-fold) upon cyanobacterium cultivation in the media with high iron concentrations, 64 mg Fe L^-1^, compared to the control Zarrouk’s medium (Fig. 1a). This high absorbed iron did not impair cyanobacterial growth, but instead increased its specific growth rate and maximum biomass (Table 1). Iron is a critical trace element for *A. platensis*, where it serves as a pivotal cofactor in photosynthesis within the electron transport chain and carbon fixation processes that collectively enhance biomass production ^15^. In agreement with other studies ^30^, iron supplementation < 10 g L^-1^ had no significant effect on the growth rate; while higher iron levels induced cell growth (Table 1). This growth enhancement is consistent with higher nutritional value because of increase in macronutrients such as carbohydrates and proteins which are essential for basic human nutrition as well as enhancement in bioactive compounds which provide health benefits ^31^.

Higher iron concentration led to higher photosynthetic performance (see the supplementary material) ^32^. The increase in total carbohydrates in response to iron concentration (Fig. 1c) can also be attributed to photosynthetic performance. However, at higher iron concentration, the ROS accumulates as well (Fig. 2a and b). The high oxidative status will inactivate the photosystem because in this case, oxygen rather than ferredoxin receive the electrons, Mehler reaction ^32^. At higher iron concentration > 16 mg L^-1^, the oxidative defense mechanism is activated, accompanied by higher antioxidant molecules such as phenolics (Fig. 2c) and carotenoids (see the supplementary material) and lower reactive oxygen species (Fig. 2a). Besides, an increase in ROS activates several metabolic pathways such as starch biosynthesis through enhanced activation of ADP-glucose pyrophosphorylase, sucrose metabolism ^33^, and pentose phosphate pathway ^34^ as well as regulate hormonal synthesis such as abscisic acid which led to carbohydrate accumulation in response to stress ^35^. These phenomena possibly caused almost constant total carbohydrates upon exposure to iron concentrations ranged from 8 to 32 mg L^-1^, followed by a significant increase (> 14%) at 64 mg Fe L^-1^ than lower levels.

Cellular protein content follows iron content in the media. Because of iron redox activity and cellular homeostasis requirement, iron was found in trivalent state in the storage molecule ferritin or divalent state in transport^15^. Accordingly, an increase in total protein (Fig. 1b) can be attributed to the key role of ferric iron transport system which involves an iron binding protein FutA2, a peripheral membrane ATPase FutC, and intracellular protein FutA12. Also, the cyanobacterium produces higher ferritin protein to bind intracellular storage iron and to cope with potential cell damage under high oxidative status (Fig. 2a and b). Total protein of *A. platensis* was increased with higher iron concentrations in the culture media. Similar results have been observed in *A. platensis* and other cyanobacteria, indicating a positive relationship between iron availability and the enhancement of these biomolecules^3,36^. The combination of increased iron bioaccumulation and protein content highlights the potential of *A. platensis* as a nutritionally enhanced food supplement.

Additionally, the fatty acid profile of *A. platensis* contributes to its nutritional value. Several key fatty acids such as palmitic, oleic, stearic, palmitoleic, linoleic, and γ-linolenic acids have been found in the lipid profile ^1,37,38^. Variations in this profile often result from differences in the proportions of saturated and unsaturated fatty acids, which are directly influenced by growth conditions. In this study, *A. platensis* cultured under varying iron concentrations exhibited notable changes in its fatty acid profile (Table 2) in agreement with previous findings. For instance, earlier studies have reported that maintaining FeSO₄ concentrations between 50 and 100 mg L^-1^ leads to increased levels of palmitic, oleic, γ-linolenic, and linoleic acids ^1^, while lower iron levels result in reduced fatty acid production (Table 2). A shift in fatty acid composition towards polyunsaturated fatty acids can have significant implications for the development of nutritional supplements ^39,40^. Consistent with other studies, palmitic acid (C16:0) remained the dominant saturated fatty acid across all iron concentrations, and its concentration increased directly with iron levels ^1,38^. Meanwhile, palmitoleic acid (C16:1) also increased with higher iron concentrations, peaking at 32 mg L^-1^ and potentially enhancing the nutritional quality of the biomass. At elevated iron levels, decline in stearic acid (C18:0) and oleic acid (C18:1) suggests a possible increase in the relative proportion of unsaturated fatty acids. Additionally, the significant increase in linolenic acid at 32 mg L^-1^ possibly reflects an adaptive response to iron-induced oxidative stress. In agreement with these observations, previous research has demonstrated that environmental stressors such as fluctuations in temperature and oil supplementation can promote the accumulation of gamma-linolenic acid in *A. platensis* ^41^. These observations are consistent with the higher oxidative status upon higher iron supplementation (Fig. 2). The mono-and poly-unsaturated fatty acids, in particular gamma-linolenic acid, were reported to regulate blood pressure, lower cholesterol levels, and modulate oxidative stress, helping treat cardiovascular diseases and inflammation and delay the effects of aging ^3^.

In this study, increased iron concentrations were observed to associate with a higher oxidative status, consistent with previous research ^15^. Notably, MDA levels in *A. platensis* was detected to increase significantly with higher iron concentrations, reaching a peak at the highest concentration 64 mg L^− 1^ (Fig. 2b). This data suggests that increased iron availability may impair nutritional value because of the formation of free radicals which can damage cellular components and disrupt normal metabolic processes. However, the moderate iron supplementation not only enhanced growth rate and dry biomass of *A. platensis*, but also added to its nutritional value through enhanced production of bioactive organics. Under environmental stress conditions, *A. platensis* and other algae have sophisticated defense mechanisms to mitigate damage. One key strategy is the synthesis of PBPs, a type of powerful antioxidant that can comprise up to half of the total soluble cellular proteins. These pigments are found in phycobilisomes on the outside of chloroplast thylakoids, where they absorb solar energy for photosynthesis and also protect cells from ROS generated under stress conditions. It should be noted that *A. platensis* is renowned for its exceptionally high C-phycocyanin (C-PC) content. While some studies report that stress condition can boost carotenoid and PBP levels to mitigate oxidative damage, other research indicates that excessive iron availability may adversely affect the production of phycocyanin, proteins, and lipids ^2,6,30^. These findings reinforce the present observation that optimal iron levels are critical for balancing pigment synthesis and antioxidant defense. In this study, we observed that the levels of PBPs in *A. platensis* were significantly increased by moderate levels of iron in the culture media (Table 3), with PC, APC, and PE reaching their highest levels at moderate iron concentrations of 16–32 mg L^[− 1 2,30^. This suggests that while iron is essential for pigment synthesis, excessive iron may not further enhance pigment production, possibly due to metabolic shifts or the activation of stress responses. These pigments are health promoting compounds which demonstrated anti-cancer, anti-inflammatory, anti-hypertensive, neuroprotective, and immune-modulating effects ^3,42^. Considering the yield of these pigments, especially PC (Table 3), the daily consumption of the fortified spirulina biomass (~ 3 g) can supply the recommended daily dose of > 100 mg pure PC ^43,44^.

Additionally, *A. platensis* contains several phenolic acids such as gallic acid, hydroxybenzoic acid, ferulic acid, salicylic acid, benzoic acid, and cinnamic acid. The measured values of phenolic acids in the present study were consistent with the values reported in other studies^42^. These compounds are complex molecules characterized by an aromatic ring with one or more hydroxyl groups, and they possess several important properties, most notably antioxidant activities^31,45^. Phenolic compounds are also essential for regulating stress tolerance. Under stress conditions, cyanobacteria increase the production of these compounds, which aids in their adaptability and boosts their overall resilience. These metabolites contribute to stress protection by chelating metal ions, thus reducing metal toxicity and oxidative damage^46^. Total phenolics in *A. platensis* is dynamic and also influenced by environmental conditions, growth stage, and the nutritional composition of the culture medium^45,47^. Similarly, varying iron concentrations significantly impacted the accumulation of total phenolics, with the highest content at 64 mg L^− 1^ (Fig. 2c). The highest phenolics at the highest iron content indicating that iron availability may stimulate phenolic biosynthesis or enhance the organism’s antioxidative response.

These findings align with other research findings, suggesting that moderate to elevated levels of certain micronutrients can upregulate secondary metabolite production in cyanobacteria^48^. A positive relationship between iron biofortification and phenolic synthesis was observed in this work (Fig. 2c). The phenolic profile analysis was also revealed notable increases in specific compounds, particularly benzoic acid, salicylic acid, and gallic acid (Table 4). The phenolics account for approximately 99% of the observed antioxidant activity, underscoring their critical role in maintaining cellular health and supporting the therapeutic potential of *A. platensis*^49^. For instance, benzoic acid is recognized for its strong antioxidant and anti-inflammatory properties^50,51^. Gallic acid has been shown to enhance antioxidative defense mechanisms by reducing ROS and boosting antioxidant enzyme activity^52^. In contrast, ferulic acid reached its maximum at 8 and 16 mg L^− 1^ but declined at elevated iron levels, suggesting possible feedback inhibition or metabolic rerouting toward other phenolic-related pathways. Salicylic acid remained relatively stable, slightly increased, with increasing iron concentration, implying that its biosynthesis may be independent of iron availability. Interestingly, salicylic acid modulates folate accumulation in plants by regulating gene expression and protein activity, highlighting its role in metabolic pathways^53^. Cinnamic acid levels also remained unchanged, indicating a minimal impact of iron concentration on its production. These findings suggest the complex interplay between iron supplementation and phenolic metabolism in *A. platensis*. Overall, the data suggest that each phenolic compound responds differently to varying iron concentrations, highlighting the importance of optimizing iron levels to achieve desired phenolic profiles and potentially enhance the organism’s nutraceutical value^15^.

Phenolic compounds function as potent antioxidants by acting as metal chelators, hydrogen donors, and free radical scavengers, thereby bolstering cellular antioxidant defenses^47,49,54^. Moreover, phenolic compounds serve as potent antioxidants by binding to metal ions such as Fe and Cu, which are known to promote the formation of free radicals. On the other hand, very high phenolics are not suitable because of their anti-nutritional role and potential to inhibit iron absorption in small intestine. Besides, high iron content can increase the risk of iron-induced oxidative stress^15^. These findings support the notion that the moderate levels of these phenolic acids in *A. platensis*, as a result of iron biofortification, can enhance its functional properties and further establish its value as a health-promoting dietary supplement. Considering the recommended daily uptake of 3–10 g spirulina biomass^55^, the moderate levels of iron treatment can provide us with the required 30 mg iron per day (Fig. 1).

Consequently, these findings underscore the suitability of *A. platensis* for iron biofortification and the development of iron-dense supplements^4,15^. Several studies have reported that *A. platensis* supplementation can improve hemoglobin levels and replenish iron store^2,30^. Building on these findings, the current study explored iron biofortification in *A. platensis* is possible without any adverse effects on the biochemical composition.

Further studies on the profile of essential amino acids under varying iron levels would be valuable for elaborating on the present findings for the transformative role of iron biofortification. It is also suggested that future studies assess the iron release and bio-accessibility in the human gastrointestinal tract using in vitro and in vivo models.

## Conclusion

The complex interplay between iron concentration, oxidative stress, and antioxidant defense in *A. platensis* caused significant changes in the biomass productivity and biochemical composition. The moderate iron levels (16–32 mg L^-1^) enhanced the biomass growth rate possibly due to improved photosynthetic performance, while higher levels impaired growth because of oxidative stress, resulting in constant biomass productivity despite 30-fold higher total absorbed iron. The total proteins and carbohydrates were monotonically increased with iron levels. The highest PUFAs and PBPs, particularly PC, were observed at moderate iron levels. In contrast, higher iron levels enhanced ROS generation, consequently shifted the antioxidant defense mechanism towards enhanced phenolic production, in particular gallic acid and benzoic acid, and possibly compromised the bio-accessibility of absorbed iron which need to be assessed in the future research. Thus, an optimal iron concentration is critical to maximize the productivity and nutritional value of spirulina biomass.

## Supplementary Information

Below is the link to the electronic supplementary material.

Supplementary Material 1

## Data Availability

All data generated or analyzed during this study are included in this article and its Supplementary Information file.

## Abbreviations

APC: Allophycocyanin
P_max_: Biomass productivity
X _max_: Dry biomass
Dt: Doubling time
GC: Gas chromatography
GAE: Gallic acid equivalent
HPLC: High-performance liquid chromatography
H_2_O_2_: Hydrogen peroxide
OH^·^: Hydroxyl radicals
IDA: Iron deficiency anemia
PBPs: Phycobiliproteins
PC: Phycocyanin
PE: Phycoerythrin
ROS: Reactive oxygen species
µ_max_: Specific growth rate
O^2−^: Superoxide anion
TBA: Thiobarbituric acid
TPC: Total phenolic content
TCA: Trichloroacetic acid

## Declarations

**Statement**.
