# Supplementary material for: Iron biofortification as a promising strategy to improve productivity and nutritional value of Arthrospira platensis (spirulina)
Source: Sci Rep. 2026 Feb 21;16:10099. doi: 10.1038/s41598-026-40520-8 (PMC13022260; doi:10.1038/s41598-026-40520-8)
Supplement: Supplementary file 1 — Supplementary Material 1 [file 41598_2026_40520_MOESM1_ESM.docx]

**Iron Biofortification as a Promising Strategy to Improve Productivity and Nutritional Value of *Arthrospira platensis* (spirulina)**

Fatemeh Gholizadeh^a^, Fatemeh Zarinkamar ^a*^

^a^Department of Plant Biology, Faculty of Biological Sciences, Tarbiat Modares University, Tehran, Iran

*Corresponding author: Fatemeh Zarinkamar, PhD, Email: [zarinkamar@modares.ac.ir](mailto:zarinkamar@modares.ac.ir)

**Figure S1** the growth curve of *Arthrospira platensis* in the modified Zarrouk’s medium supplemented with varying iron concentrations from 2 (control) to 64 mg L^-1^

**
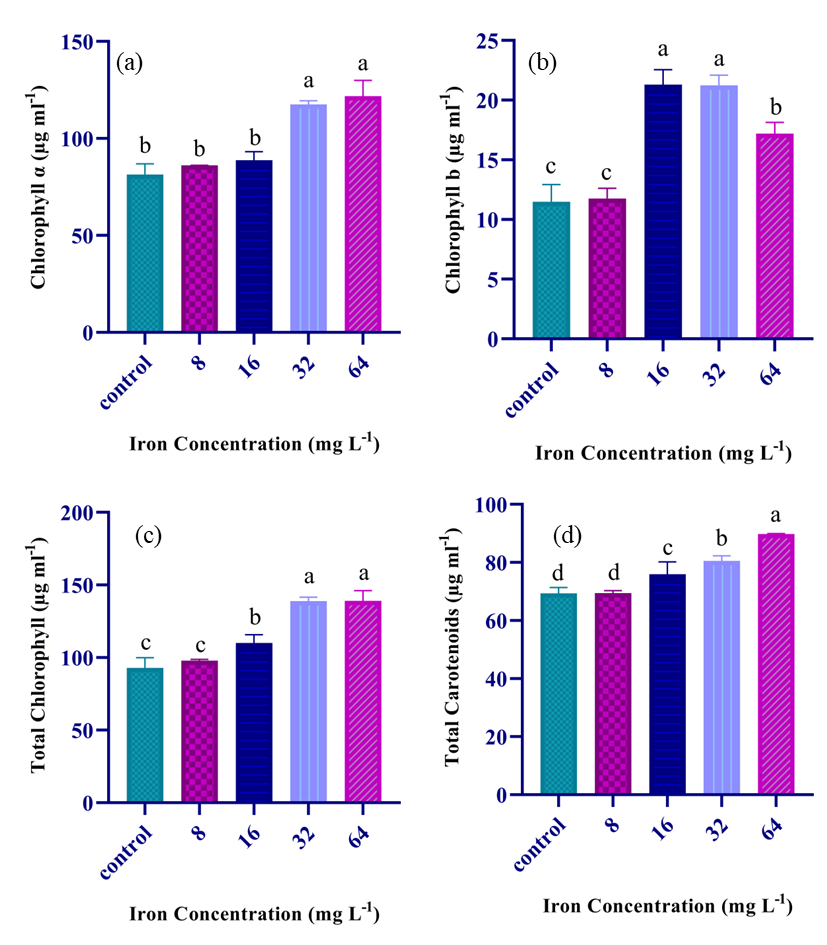
**

**Figure S2** Effect of varying iron concentrations in the culture media on the *chl a* (a), *chl b* (b), total chl (c) and total carotenoids (d) of *Arthrospira platensis*
